# Early detection of heart function abnormality by native T1: a comparison of two T1 quantification methods

**DOI:** 10.1007/s00330-019-06364-9

**Published:** 2019-08-13

**Authors:** Pandji Triadyaksa, Dirkjan Kuijpers, Tugba Akinci D’Antonoli, Jelle Overbosch, Mieneke Rook, J. Martijn van Swieten, Matthijs Oudkerk, Paul E. Sijens

**Affiliations:** 1 University of Groningen, Hanzeplein 1, Groningen, 9713 GZ The Netherlands; 2 Department of Physics, Diponegoro University, Prof. Sudharto street, Semarang, 50275 Indonesia; 3 Department of Radiology, HMC-Bronovo, Bronovolaan 5, The Hague, 2597 AX The Netherlands; 4 University Hospital Basel, Clinic of Radiology & Nuclear Medicine, University of Basel, Petersgraben 4, 4031 Basel, Switzerland; 5 Department of Radiology, University Medical Center Groningen, Hanzeplein 1, Groningen, 9713 GZ The Netherlands; 6 Institute for Diagnostic Accuracy, Groningen, The Netherlands; 7 Department of Radiology, EB45, University Medical Center Groningen, P.O. Box 30001, 9700 RB Groningen, The Netherlands

**Keywords:** Magnetic resonance imaging, Myocardium, Cardiomyopathies, Statistical distribution

## Abstract

**Objective:**

To compare the robustness of native T1 mapping using mean and median pixel-wise quantification methods.

**Methods:**

Fifty-seven consecutive patients without overt signs of heart failure were examined in clinical routine for suspicion of cardiomyopathy. MRI included the acquisition of native T1 maps by a motion-corrected modified Look-Locker inversion recovery sequence at 1.5 T. Heart function status according to four established volumetric left ventricular (LV) cardio MRI parameter thresholds was used for retrospective separation into subgroups of normal (*n* = 26) or abnormal heart function (*n* = 31). Statistical normality of pixel-wise T1 was tested on each myocardial segment and mean and median segmental T1 values were assessed.

**Results:**

Segments with normally distributed pixel-wise T1 (57/58%) showed no difference between mean and median quantification in either patient group, while differences were highly significant (*p* < 0.001) for the respective 43/42% non-normally distributed segments. Heart function differentiation between two patient groups was significant in 14 myocardial segments (*p* < 0.001–0.040) by median quantification compared with six (*p* < 0.001–0.042) by using the mean. The differences by median quantification were observed between the native T1 values of the three coronary artery territories of normal heart function patients (*p* = 0.023) and insignificantly in the abnormal patients (*p* = 0.053).

**Conclusion:**

Median quantification increases the robustness of myocardial native T1 definition, regardless of statistical normality of the data. Compared with the currently prevailing method of mean quantification, differentiation between LV segments and coronary artery territories is better and allows for earlier detection of heart function impairment.

**Key Points:**

• *Median pixel-wise quantification of native T1 maps is robust and can be applied regardless of the statistical distribution of data points.*

• *Median quantification is more sensitive to early heart function abnormality compared with mean quantification.*

• *The new method yields significant native T1 value differentiation between the three coronary artery territories.*

**Electronic supplementary material:**

The online version of this article (10.1007/s00330-019-06364-9) contains supplementary material, which is available to authorized users.

## Introduction

Pre-contrast T1 relaxation time, the parameter at stake in native T1 mapping, has shown its potential for identifying myocardial tissue abnormality [1], with the limitation that the values measured are sequence-specific [2–7]. Native T1 increases may indicate disease and have been associated with diffuse myocardial fibrosis in different types of cardiomyopathy [7–15]. Moreover, in patient groups with myocardial impairment, an increase of native T1 was observed in the absence of late gadolinium enhancement (LGE) [7, 9, 10, 14, 15] suggesting that native T1 mapping can be an early indicator of myocardial tissue abnormality. Therefore, a robust native T1 quantification method is needed to ensure early identification of heart function abnormality.

**Fig. 1:**
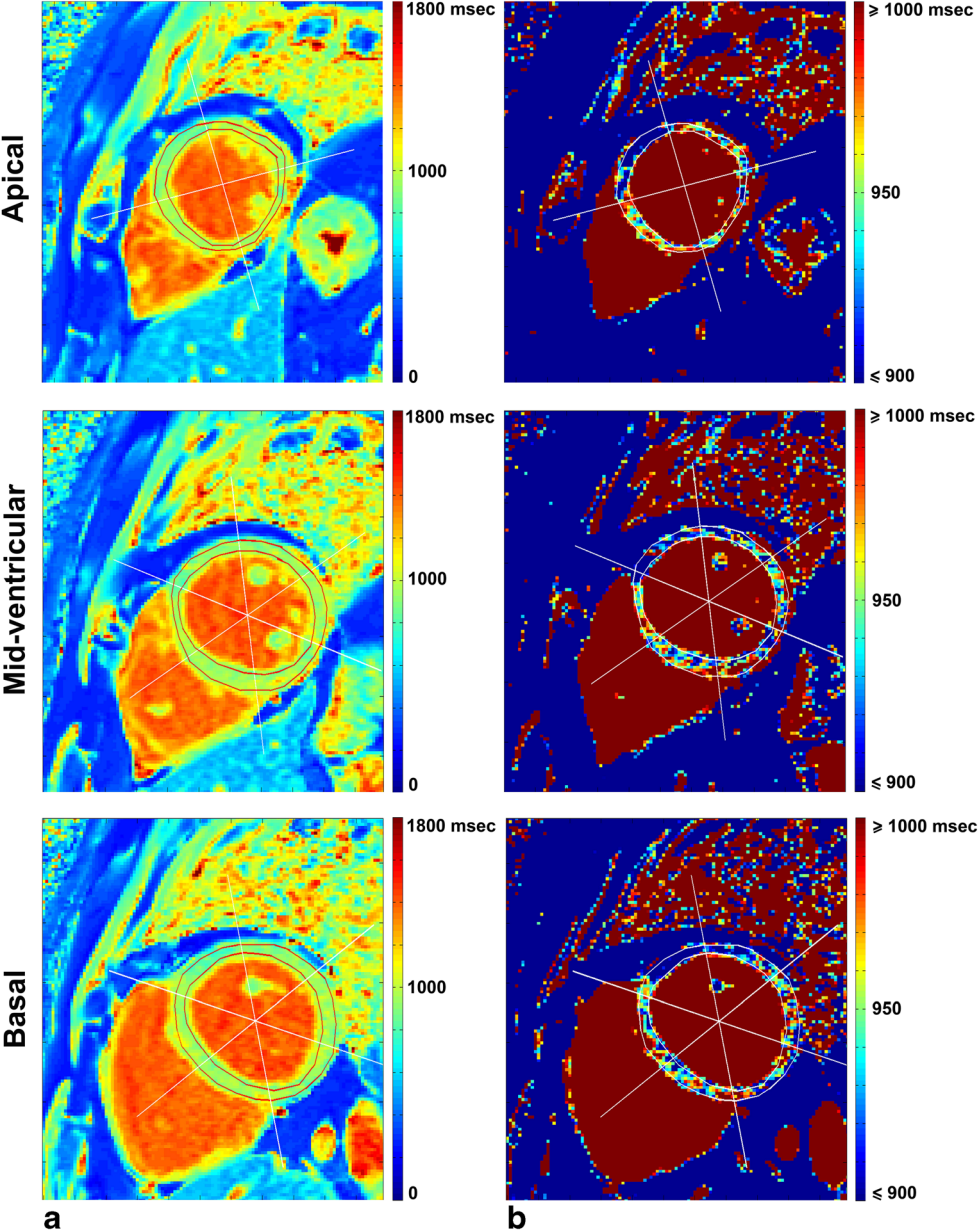
Native T1 mapping of the left ventricular myocardium, three short-axis slices segmented by the AHA model in a case of normal heart function scaled (**a**) from 0 to 1800 ms and (**b**) from 900 to 1000 ms to show T1 heterogeneity

In measuring cardiac T1 value, numerous studies showed normal native T1 variation on different myocardial regions [4, 5, 8, 10, 11, 13, 16–19]. Intersegmental variations complicate the standardisation of normal values and disease identification. Pixel-wise T1 value quantification also faces variability due to protocol parameters, sequence design, scanner adjustment, T1 fit model, tissue characteristics, and patient’s condition [6, 20]. In view of the heterogeneity of pixel-wise T1 values as illustrated in Fig. 1, variability may be reduced by the assessment of median values of pixel-wise T1 per segment rather than the evaluation of the means [14].

In liver and heart iron deposition assessment by T2* mapping, pixel-wise median quantification produced lower observer variability compared with mean quantification [21] and lower T2* variability in different myocardial regions [22, 23]. These studies showed that partial volume effect, heart motion artefact, the fitting model used, and observer’s myocardial contour determination influence the pixel-wise assessment and quantification in the region of interest. However, pixel-wise native T1 assessment studies published to date used mean quantification with a few ones checking the normality of the statistical distribution of datasets as a whole [11, 13–15, 24] rather than performing statistical normality testing of pixel-wise T1 distribution per segment. This study aims to investigate the normality of pixel-wise T1 values per left ventricular heart segment and subsequently compare the mean and median values. Application of both methods on patients with normal and abnormal heart function is used to assess their potential for early detection of heart function abnormality.

## Materials and methods

This retrospective analysis was conducted on magnetic resonance imaging (MRI) data acquired from May until October 2015 with approval by the hospital review board waiving the requirement of informed consent. MRI including (native) T1 mapping sequences was used to evaluate 145 consecutive patients examined in clinical routine for suspicion of cardiomyopathy. Patients with overt signs of heart failure, i.e. LGE pattern (observed 10–15 min after 0.2 mmol/kg of gadoterate meglumine: Dotarem, Guerbet), irregular heartbeat or myocardial wall, and cavum thickening, were excluded. The remaining 57 patients were divided into two groups with either normal or abnormal functional heart magnetic resonance (MR) parameters. Normal heart function was defined as three of four MR parameters (i.e. left ventricle (LV) end-diastolic volume, LV end-systolic volume, stroke volume, and ejection fraction) being within the normal MR parameter ranges and the fourth still within the border line of normality as defined by Kawel-Boehm et al [25].

### Cardiac magnetic resonance imaging

All MR scans were performed on a 1.5-T whole-body scanner (Aera, Siemens Medical Solutions). Functional heart MR parameters were acquired by performing cine imaging steady-state free precession images with echo time (TE) 1.1 ms, repetition time (TR) 42.1 ms, flip angle (FA) 56°, reconstructed voxel size 1.82 × 1.82 × 8 mm, field of view (FOV) 349 × 349, matrix 192 × 192, pixel bandwidth 930 Hz, phase resolution sampling 70%, phase FOV 100%, and GeneRalized Autocalibrating Partial Parallel Acquisition (GRAPPA) acceleration factor 2.

Modified Look-Locker inversion recovery (MOLLI) was implemented in a single breath hold at late diastole, using vendor-provided motion correction for T1 mapping based on image registration with synthetic image estimation [26]. The 5(3)3 MOLLI protocol acquired 5 images after the first inversion pulse, followed by a pause of 3 heartbeats prior to the acquisition of the next 3 images after the second inversion pulse. The protocol’s initial inversion time (TI) was 100 ms, TE 1.12 ms, TR 280.56 ms, and FA 35°. Reconstructed voxel size was 1.41 × 1.41 × 8 mm, FOV 306 × 360, matrix 218 × 256, phase resolution sampling 66%, phase FOV 85%, and GRAPPA acceleration factor 2.

### Image analysis

T1 maps were generated by custom-written software (developed in MATLAB version 7.14, The MathWorks) at three short-axis locations (apical, mid-ventricular, and basal) using pixel-wise fitting of a three-parameter model [20]:

1 $$ SI=A-B{e}^{-\raisebox{1ex}{$ TI$}\!\left/ \!\raisebox{-1ex}{$T{1}^{\ast }$}\right.} $$

to acquire *T*_1_ as:

2 $$ T1=T{1}^{\ast}\left(\raisebox{1ex}{$B$}\!\left/ \!\raisebox{-1ex}{$A$}\right.-1\right) $$

where SI and TI are signal intensity and inversion time, respectively, while *A* and *B* are constant values. Two cardiac radiologists (with 5 and 7 years of experience, respectively) and two non-cardiac experts (a radiology technician with 15 years of experience and a non-cardiac radiologist with less than 1 year of experience in cardiac imaging) manually drew LV endocardial and epicardial contours once on the T1 map while carefully avoiding LV blood pool and epicardial fat (Fig. 1). Segmental T1 analysis was conducted on all pixels (without applying endocardial/epicardial inset correction) according to the American Heart Association (AHA) 16-segment model [19] on global myocardium by averaging the 16 segments, different slice locations, and different coronary artery territories [27].

The volumetric cardiac MR parameters were evaluated by a cardiac imaging post-processing radiology technician using QMASS software (Medis Medical Imaging Systems) and checked by a cardiac radiologist (Table 1).

**Table 1:** Characteristics of patients with normal and abnormal heart function according to the criteria of Kawel-Boehm et al [24]

	Normal heart function (*n* = 26)	Abnormal heart function (*n* = 31)	*p* value^a^
General parameter			
Number of males	13 (50)^b^	17 (55)^b^	0.716^c^
Age (years)	47 ± 19	41 ± 18	0.279^d^
Heart rate (bpm)	67 ± 8	66 ± 7	0.706
BMI (kg/m^2^)	25.15 ± 2.50	24.00 ± 2.60	0.481
BSA (m^2^)	1.96 ± 0.22	1.97 ± 0.25	0.940^d^
MR measured parameter			
LV mass (g)	86.67 ± 20.47	105.04 ± 22.14	0.031
LV mass index (g/m^2^)	44.11 ± 10.42	53.34 ± 11.24	0.033
LV EDV (ml)	155.97 ± 20.52	214.44 ± 25.91	< 0.001
LV EDV index (ml/m^2^)	79.39 ± 10.44	108.89 ± 13.16	< 0.001
LV ESV (ml)	61.23 ± 11.46	101.93 ± 22.48	< 0.001
LV ESV index (ml/m^2^)	31.16 ± 5.83	51.76 ± 11.41	< 0.001
Stroke volume (ml)	96.76 ± 11.58	104.55 ± 16.23	0.305
LV EF (%)	61.50 ± 3.87	49.50 ± 6.15	< 0.001^d^
Cardiac output (L/min)	6.14 ± 1.13	6.50 ± 1.21	0.773

Values are presented as mean ± standard deviation or median ± median absolute deviation or n (%). *n*, number of patients; *bpm*, beats per minute; *BMI*, body mass index; *BSA*, body surface area; *MR*, magnetic resonance; *LV*, left ventricle; *EDV*, end diastolic volume; *ESV*, end systolic volume; *EF*, ejection fraction

^a^*p* values calculated by the Mann-Whitney U test

^b^Value is number of patients, with percentage in parentheses

^c^*p* value by the chi-square test

^d^*p* values by the independent *t* test

### Statistical analysis

Statistical normality testing of data distribution was assessed using the Shapiro-Wilk test using custom-written software (developed in MATLAB version 7.14) [28]. Cardiac MR parameter of a dichotomous variable was compared using the chi-square test and continuous variables were compared using independent *t* test or the Mann-Whitney U test as appropriate. On normal and abnormal heart function patient groups, each segment T1 quantification was reported both using mean ± standard deviation (SD) and median ± median absolute deviation (MAD) [29, 30] regardless of segment’s statistical normality status. On segments having normally distributed and non-normally distributed pixel-wise T1, comparison between mean and median T1 quantification was assessed by the Mann-Whitney U test. The agreements between mean and median segmental T1 quantification were assessed using the Bland-Altman plot with a limit of agreement (LoA) set to be 1.96 × SD of the difference.

A coefficient of variance (CoV) of the T1 relaxation time was calculated as the SD of the difference divided by the mean and expressed in percentage. Comparison of T1 values between two patient groups on different LV regions was conducted using the independent sample *t* test for data evaluated by the mean and the Mann-Whitney U test for data evaluated by the median. Multiple comparisons across myocardial regions were done by the Kruskal-Wallis test with the Dunn-Bonferroni post hoc test adjustment. Statistical analyses were performed using IBM SPSS statistics software version 23 (IBM Corporation) with *p* < 0.05 considered as statistically significant.

## Results

### Patient classification

According to the criteria of Kawel-Boehm et al [20], 26 of 57 patients were classified in normal heart function group and the remaining 31 patients were classified in abnormal heart function group with similar general characteristics, such as age, heart rate, body surface area, and body mass index (*p* > 0.05). Their characteristics are listed in Table 1 (and differentiated by gender, in Supplementary Table S1).

### Statistical normality of native T1 data distribution

The assessment of AHA 16 segments of LV myocardium from 26 normal patients and 31 abnormal heart function patients resulted in a total of 416 and 496 segments, respectively. With four observers assessing these segments, we obtained 1664 and 1984 segments, respectively.

In all segments of normal patients, statistical normality testing of pixel-wise native T1 per segment showed that 964 of 1664 segments (58%) were statistically non-normally distributed, whereas in all segments of abnormal patients, this statistical distribution was found in 1140 of 1984 segments (57%). In segments having statistically normally distributed pixel-wise T1 (subject for mean quantification), segmental T1 quantification by either mean or median showed no significant difference of T1 value in normal heart function group (*p* = 0.532) and in abnormal heart function group (*p* = 0.628). This indicates that in statistically normally distributed data, median quantification is equivalent to the use of the mean. For segments with non-normally distributed pixel-wise T1 (subject for median quantification), a significant difference was found between the two T1 quantifications in both normal (*p* < 0.001) and abnormal heart (*p* = 0.003) function groups. This finding indicates that mean quantification cannot be used for statistical non-normally distributed data.

**Fig. 2:**
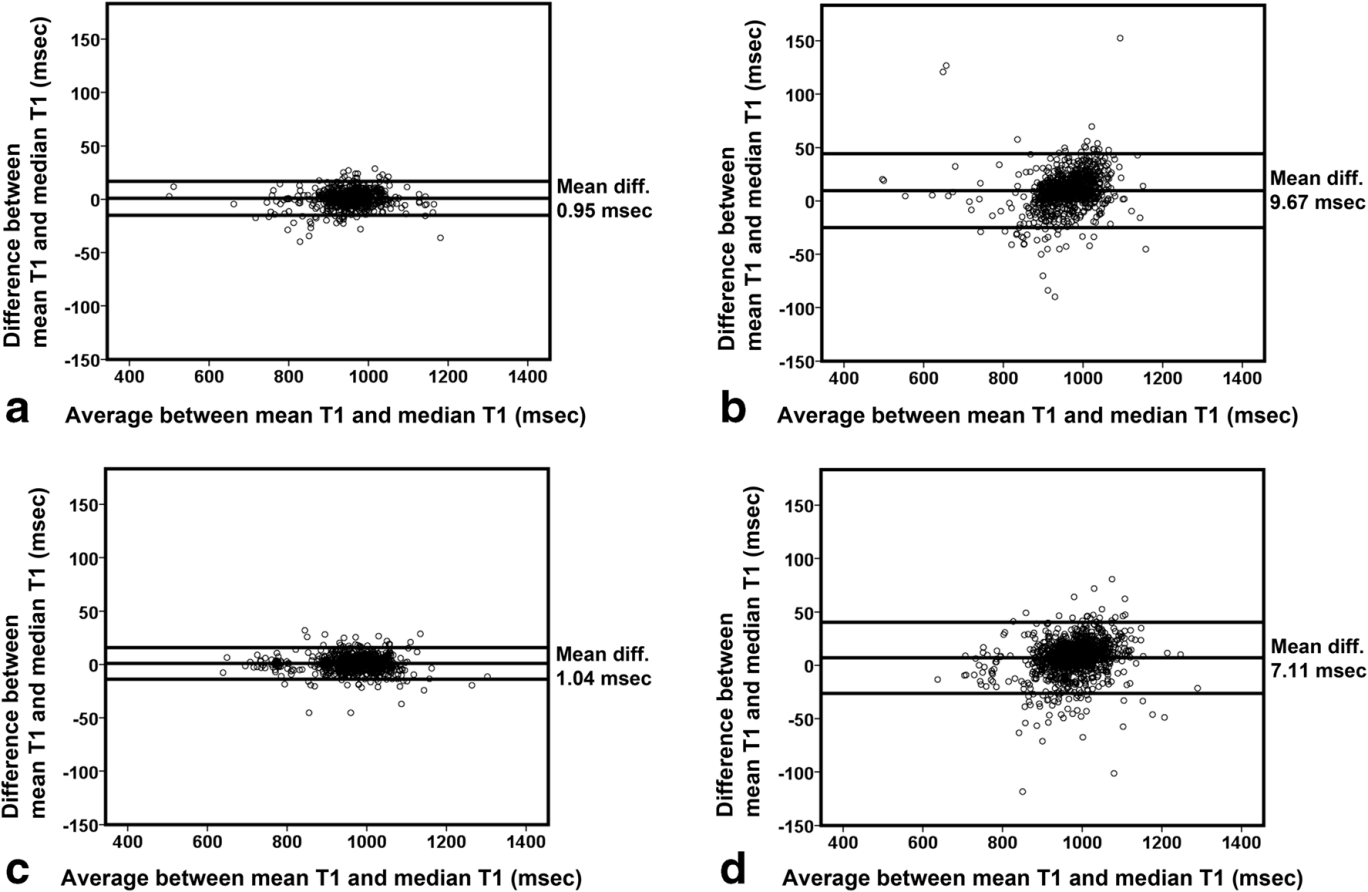
Bland-Altman plot assessment of pixel-wise native T1 agreement per segment quantified by means and medians. **a** Quantification, in normal heart function patients, for segments having statistical normally distributed and statistical non-normally distributed pixel-wise T1 (**b**). **c** Quantification, in abnormal heart function patients, for segments having statistical normally distributed and statistical non-normally distributed pixel-wise T1 (**d**)

The Bland-Altman plot confirms these claims in normal heart function patients by showing smaller differences of pixel-wise T1 assessed by mean and median quantification for segments having statistically normally distributed pixel-wise T1 (mean difference of 0.95 ms, CoV of 0.85%, and LoA of 15.96 ms) (Fig. 2a) compared with segments with non-normally distributed T1 (mean difference of 9.67 ms, CoV of 1.84%, and LoA of 34.72 ms) (Fig. 2b). Likewise, in abnormal heart function patients (Fig. 2c), pixel-wise T1 had similar smaller Bland-Altman mean difference of 1.04 ms, CoV of 0.78%, and LoA of 14.83 ms in statistically normally distributed data as opposed to higher Bland-Altman of (mean differences of 7.11 ms, CoV of 1.74%, and LoA of 33.39 ms) in non-normally distributed data (Fig. 2d).

### Regional T1 analysis and heart function abnormality

In a regional myocardial analysis (Table 2), improvement of interobserver reproducibility of segmental T1 values was found for most regional myocardium areas in normal and abnormal heart function patients when using median compared with the mean for its pixel-wise quantification. This was indicated by CoV reductions, whereas results were similar for observers with different cardiac imaging expertise background (Supplementary Table S2).

**Table 2:** T1 coefficient of variance between all observers in different left ventricular myocardial regions

			Patients with normal heart function			Patients with abnormal heart function	
			CoV between observers using			CoV between observers using	
		ns^a^	Mean T1	Median T1	ns^a^	Mean T1	Median T1
Global LV myocardium		2496	5.29	4.88	2976	4.31	3.62
LAD		936	5.33	4.73	1116	4.52	3.59
RCA		780	4.18	3.64	930	3.85	3.12
LCx		780	6.17	6.04	930	4.42	4.05
Basal		936	3.60	2.88	1116	3.29	2.56
1	Anterior	156	4.25	2.73	186	4.71	3.54
2	Anteroseptal	156	2.82	2.40	186	2.32	1.80
3	Inferoseptal	156	2.83	2.50	186	2.45	2.01
4	Inferior	156	2.68	1.78	186	3.34	2.71
5	Inferolateral	156	2.95	2.07	186	2.85	2.21
6	Anterolateral	156	5.12	4.65	186	3.39	2.58
Mid-ventricular		936	5.12	4.52	1116	4.08	3.28
7	Anterior	156	6.37	6.02	186	5.96	4.70
8	Anteroseptal	156	4.04	3.15	186	3.30	2.43
9	Inferoseptal	156	1.91	1.43	186	2.50	1.78
10	Inferior	156	3.44	2.19	186	3.27	2.91
11	Inferolateral	156	5.62	5.00	186	3.39	2.73
12	Anterolateral	156	7.27	6.83	186	4.85	4.09
Apical		624	7.40	7.32	744	5.75	5.14
13	Anterior	156	8.86	8.52	186	6.35	5.33
14	Septal	156	3.34	2.48	186	2.77	2.26
15	Inferior	156	7.83	7.33	186	6.39	5.09
16	Lateral	156	8.53	9.16	186	6.57	6.78

Data are in percentage. *LV*, left ventricle; *LAD*, left anterior descending; *RCA*, right coronary artery; *LCx*, left circumflex artery; *ns*, number of segments; *CoV*, coefficient of variance

^a^The number of segments reflects six combinations of segment comparisons between four observers

Regional T1 analysis of four observers on different LV myocardial regions by using mean and median T1 quantification is presented in Tables 3 and 4, respectively. For each table, the statistical normality testing of its data distribution per LV myocardial region is presented by Supplementary Table S3 for native T1 quantified by its mean and by Supplementary Table S4 for native T1 quantified by its median. Tables S3 and S4 show that most of the T1 data from different myocardial regions are statistically non-normally distributed reflecting inadequate use of mean quantification in Table 3 to differentiate two different patient groups. As a result, the differentiation of T1 values between normal and abnormal heart function groups is undetected in ten of 16 AHA segments of Table 3 (*p* = 0.059–0.879). When comparing the two patient groups using median quantification (Table 4), significant increase of T1 values is identified in abnormal heart function patients compared with normal heart function in all myocardial regions (*p* < 0.001–0.024) with exception in the mid-ventricular anteroseptal (*p* = 0.110) and basal anterior segments (*p* = 0.080). Heart function differentiation between the two patient groups is thus concluded to be significant in 14 myocardial segments (*p* < 0.001–0.040) by median quantification compared with only six (*p* < 0.001–0.042) when using the mean.

**Table 3:** Mean T1 value in different left ventricular myocardial regions

		T1 value of patients with normal heart function		T1 value of patients with abnormal heart function		
		ns^a^	Mean ± SD (ms)	ns^a^	Mean ± SD (ms)	*p* value ^b^
Global LV myocardium		2496	960.69 ± 60.92	2976	976.75 ± 68.65	< 0.001
LAD		936	958.85 ± 60.73	1116	974.73 ± 71.09	< 0.001
RCA		780	973.35 ± 60.23	930	989.35 ± 64.49	< 0.001
LCx		780	950.24 ± 59.65	930	966.57 ± 67.84	< 0.001
Basal		936	975.23 ± 45.31	1116	982.36 ± 63.21	0.003
1	Anterior	156	961.18 ± 46.10	186	971.17 ± 65.11	0.099
2	Anteroseptal	156	993.88 ± 48.61	186	992.96 ± 62.64	0.879
3	Inferoseptal	156	987.97 ± 42.21	186	993.07 ± 58.92	0.353
4	Inferior	156	987.97 ± 38.09	186	996.81 ± 64.46	0.117
5	Inferolateral	156	969.59 ± 39.13	186	979.66 ± 62.96	0.072
6	Anterolateral	156	950.78 ± 40.28	186	960.47 ± 57.23	0.068
Mid-ventricular		936	961.51 ± 52.86	1116	973.36 ± 64.77	< 0.001
7	Anterior	156	940.11 ± 56.45	186	953.18 ± 70.86	0.064
8	Anteroseptal	156	973.67 ± 45.14	186	977.09 ± 60.32	0.550
9	Inferoseptal	156	980.54 ± 46.28	186	990.38 ± 57.79	0.081
10	Inferior	156	976.31 ± 50.81	186	986.47 ± 58.59	0.091
11	Inferolateral	156	961.45 ± 42.94	186	982.18 ± 62.04	< 0.001
12	Anterolateral	156	936.95 ± 57.11	186	950.85 ± 67.31	0.042
Apical		624	937.66 ± 82.09	744	973.43 ± 80.65	< 0.001
13	Anterior	156	914.57 ± 79.12	186	962.01 ± 80.23	< 0.001
14	Septal	156	969.67 ± 48.66	186	991.98 ± 76.94	0.001
15	Inferior	156	933.98 ± 90.70	186	980.04 ± 79.49	0.004
16	Lateral	156	932.43 ± 92.86	186	959.71 ± 82.10	< 0.01

*SD*, standard deviation; *ns*, number of segment; *LV*, left ventricle; *LAD*, left anterior descending; *RCA*, right coronary artery; *LCx*, left circumflex artery

^a^The number of segments reflects six combinations of segment comparisons between four observers

^b^*p* values of comparison between normal and abnormal heart function groups by independent sample *t* test

**Table 4:** Median T1 value in different left ventricular myocardial regions

		T1 value of patients with normal heart function		T1 value of patients with abnormal heart function		
		ns^a^	Median ± MAD (ms)	ns^a^	Median ± MAD (ms)	*p* value ^b^
Global LV myocardium		2496	959.85 ± 30.32	2976	974.94 ± 35.07	< 0.001
LAD		936	958.52 ± 33.29	1116	971.53 ± 35.36	< 0.001
RCA		780	971.78 ± 27.95	930	987.15 ± 37.05	< 0.001
LCx		780	952.39 ± 26.26	930	964.94 ± 33.10	< 0.001
Basal		936	966.94 ± 22.61	1116	980.79 ± 33.98	< 0.001
1	Anterior	156	964.35 ± 24.48	186	969.98 ± 33.09	0.080
2	Anteroseptal	156	976.65 ± 20.45	186	1004.20 ± 39.28	0.040
3	Inferoseptal	156	971.14 ± 19.05	186	987.85 ± 36.06	< 0.001
4	Inferior	156	981.81 ± 25.15	186	990.10 ± 39.13	0.010
5	Inferolateral	156	964.20 ± 24.18	186	981.66 ± 30.88	< 0.001
6	Anterolateral	156	957.23 ± 16.38	186	960.54 ± 28.07	< 0.001
Mid-ventricular		936	956.61 ± 31.27	1116	972.02 ± 33.83	< 0.001
7	Anterior	156	938.52 ± 39.88	186	953.70 ± 28.24	< 0.001
8	Anteroseptal	156	965.38 ± 30.20	186	972.83 ± 34.06	0.110
9	Inferoseptal	156	970.71 ± 22.85	186	982.84 ± 32.18	< 0.001
10	Inferior	156	972.89 ± 27.91	186	996.98 ± 32.71	< 0.001
11	Inferolateral	156	951.55 ± 22.85	186	981.01 ± 30.19	< 0.001
Apical		624	940.58 ± 39.64	744	971.31 ± 37.26	< 0.001
12	Anterolateral	156	932.91 ± 31.00	186	946.04 ± 33.09	0.010
13	Anterior	156	919.70 ± 41.48	186	962.45 ± 31.95	< 0.001
14	Septal	156	959.46 ± 36.14	186	980.53 ± 37.96	< 0.001
15	Inferior	156	936.24 ± 46.59	186	982.10 ± 43.85	< 0.001
16	Lateral	156	953.10 ± 36.88	186	965.16 ± 49.61	< 0.001

*MAD*, median absolute deviation; *ns*, number of segments; *LV*, left ventricle; *LAD*, left anterior descending; *RCA*, right coronary artery; *LCx*, left circumflex artery

^a^The number of segments reflects six combinations of segment comparisons between four observers

^b^*p* values of comparison between normal and abnormal heart function groups by the Mann-Whitney U test

**Fig. 3:**
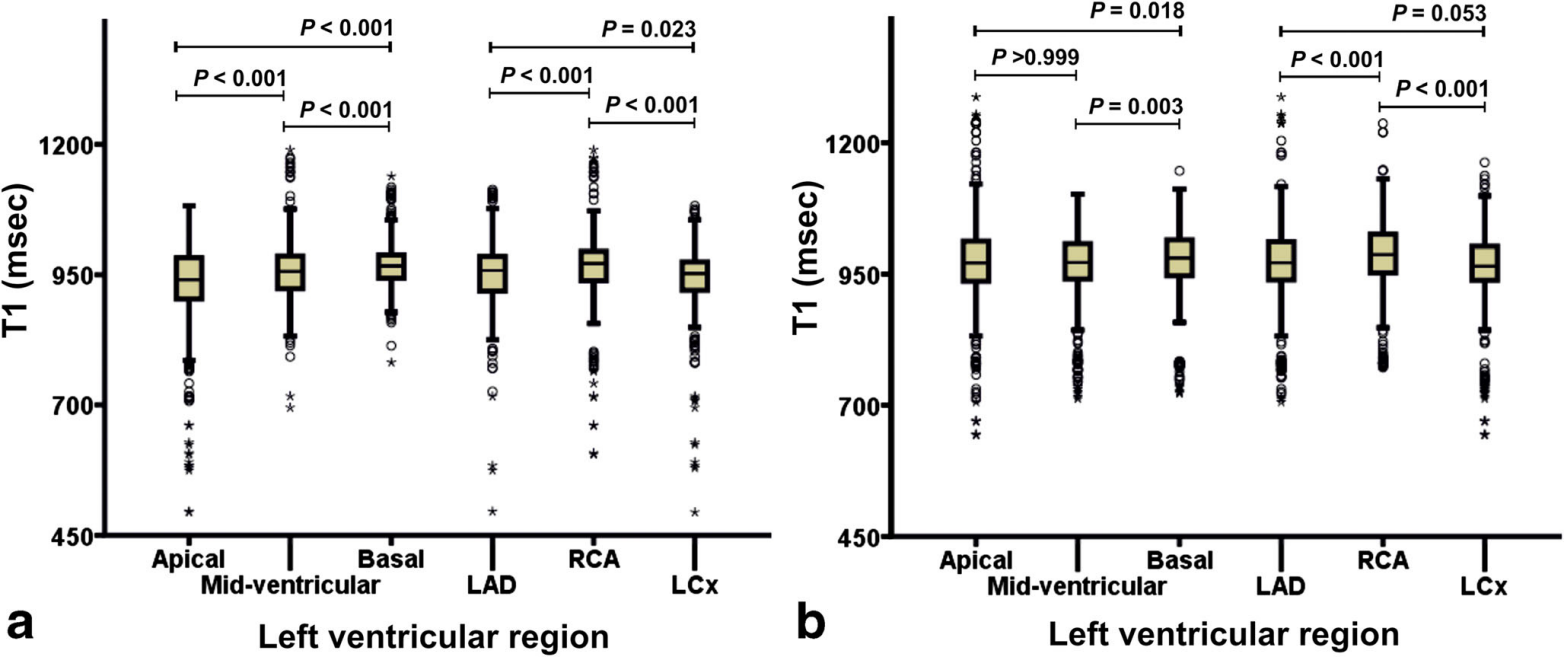
Boxplot of median T1 in different left ventricular myocardial regions. Quantifications for normal (**a**) and abnormal (**b**) heart function patients. Comparisons between the regions were made by the Dunn-Bonferroni post hoc test adjustment of the Kruskal-Wallis test result

Using median quantification, regional LV T1 value in normal heart function patient group was found to be significantly different in the three short-axis slices and in the three coronary artery territories attributed to the 16 AHA segments (Fig. 3a) (*p* < 0.001–*p* = 0.023). However, in abnormal heart function patient group (Fig. 3b), T1 value between apical vs. mid-ventricular short-axis slices and between left anterior descending (LAD) and left circumflex artery (LCx) coronary artery territories were not significantly different (*p* > 0.999 and *p* = 0.053, respectively).

## Discussion

This study shows that median value quantification can be used for segmental native T1 assessment regardless of the distribution of pixel values and therefore can replace mean value quantification where statistical data distribution is normal. Median quantification also showed robustness regardless of the observer’s background by improving interobserver reproducibility of segmental native T1. The superiority of median T1 pixel value quantification compared with mean quantification is confirmed by better differentiation observed between patients with normal and abnormal heart function, especially in the septal regions that are least sensitive to susceptibility artefacts [31]. Therefore, median quantification would be a solution to reduce the influence of any unwanted outlier pixel-wise T1 values. Another study has already promoted MAD of fitting residuals to avoid outliers in T1 fitting process yielding a robust measurement of native T1 [32].

In providing early indication of cardiomyopathy disease in patients with normal cardiac MR functional parameters, native T1 showed no value according to several studies [11, 13, 15]. Our own results obtained with statistical parametric testing and (suboptimal) mean quantification also failed to differentiate between normal and abnormal heart function patients in LV segmental native T1 evaluation. In this study, however, significant increases of T1 values in abnormal heart function patients were found when using median T1 quantification with non-parametric testing instead. Our results also suggest that parametric testing must be performed in native T1 quantification to make sure of statistical normality of the pixel-wise native T1 distribution prior to using means. Alternatively, one can simply use non-parametric testing and medians (as in this study) for the investigation of patient heart condition.

Novel findings in this study of native T1 in normal heart function patients quantified by the medians in different myocardial coronary perfusion territories (i.e. LAD, right coronary artery, LCx, apical, mid-ventricular, and basal), different short-axis slices, and different AHA segments elaborate on those in smaller studies of healthy subjects [16, 19]. The observed variation of T1 value in the LV of normal heart function patients can provide regional baseline T1 values for early detection of diffuse fibrosis and infarct identification.

Suggested elsewhere [33–36], heart wall T1 elevation is related to coronary microvascular dysfunction (CMD). Camici et al [36] explained that morphological changes of CMD in the absence of myocardial diseases are characterised by microvascular remodelling, endothelial dysfunction, and smooth muscle dysfunction. In patients developing hypertrophic cardiomyopathy, remodelling of intramural coronary arterioles will result in medial and intimal wall thickening [36]. This study reported the elevation of native T1 values in different LV regions of abnormal functional heart patients. Moreover, the variation of native T1 value observed in normal patients between LAD and LCx coronary artery territories was absent in abnormal function heart patients, an observation that might indicate early progression of CMD. But to validate this relationship, more invasive and non-invasive clinical assessment is needed and therefore recommended for further study.

Limitations of this study are that it is retrospective and that patient separation into those having a normal heart function and those without a normal heart function was based on the cardiac MR functional parameters defined by thresholds of just one reported study [25], being, however, very similar to those reported elsewhere [5, 8–12, 14, 15, 37–39]. The advice of some [17, 19, 40, 41], to correct native T1 for blood pool, heart rate, age, and gender were not followed through in this study due to the low correlation of T1 with any of these factors. Furthermore, the changes in native T1 values after correction were small and population-dependent (results not shown). Moreover, previous studies reported conflicting findings with regard to these factors’ influence on native T1 value [10, 11, 18, 39, 42]. The T1 maps generated by custom-written software yielded slightly lower values with reduced deviations for all AHA segments compared with the values produced by the Siemens Solution T1 maps (Supplementary Table S5). Investigations into T1 value differences amongst different mapping procedures and into alternative calculation algorithms to improve T1 fitting accuracy [e.g. 43] were not conducted, considered beyond the scope of this study.

Some studies reported the association between diabetes mellitus and the progression of CMD [36, 44, 45]. Another limitation of this study is that diabetes mellitus status of the patients was not recorded.

In conclusion, T1 assessment by observations of medians showed higher interobserver reproducibility compared with mean T1, regardless of statistical normality of data. Increased robustness of myocardial native T1 assessed by pixel-wise medians thus facilitates the early detection of heart function impairment and of differences between LV segments and between the different coronary artery territories.

## Electronic supplementary material

ESM 1 (DOCX 56.9 kb)

## Abbreviations

AHA: American Heart Association
CMD: Coronary microvascular dysfunction
CoV: Coefficient of variance
FA: Flip angle
FOV: Field of view
GRAPPA: Generalized autocalibrating partial parallel acquisition
LAD: Left anterior descending
LCx: Left circumflex artery
LGE: Late gadolinium enhancement
LoA: Limit of agreement
LV: Left ventricle
MAD: Median absolute deviation
MOLLI: Modified Look-Locker inversion recovery
MR: Magnetic resonance
MRI: Magnetic resonance imaging
SD: Standard deviation
TE: Echo time
TR: Repetition time
