# Supplementary material for: Early detection of heart function abnormality by native T1: a comparison of two T1 quantification methods
Source: Eur Radiol. 2019 Aug 13;30(1):652–62. doi: 10.1007/s00330-019-06364-9 (PMC6890701; doi:10.1007/s00330-019-06364-9)
Supplement: Supplementary file 1 — (DOCX 56.9 kb) [file 330_2019_6364_MOESM1_ESM.docx]

**Supplementary**

**Table S1.** Characteristics of patients with normal and abnormal heart function according to the criteria of Kawel-Boehm et al. [24].

|  | | **Normal heart function (n = 26)** | **Abnormal heart function (n = 31)** | **P-Value^a^** |
| --- | --- | --- | --- | --- |
| General parameter | |  |  |  |
|  | Number of males | 13 (50)^b^ | 17 (55)^b^ | 0.716^c^ |
|  | Age (years) | 47 ± 19 | 41 ± 18 | 0.279^d^ |
|  | Heart rate (bpm) | 67 ± 8 | 66 ± 7 | 0.706 |
|  | BMI (kg/m^2^) | 25.15 ± 2.50 | 24.00 ± 2.60 | 0.481 |
|  | BSA (m^2^) | 1.96 ± 0.22 | 1.97 ± 0.25 | 0.940^d^ |
| MR measured parameter | |  |  |  |
| Male | LV mass (g) | 113.32 ± 16.64 | 127.18 ± 36.23 | 0.174 |
|  | LV mass index (g/m^2^) | 54.60 ± 8.02 | 61.08 ±17.40 | 0.202 |
|  | LV EDV (ml) | 176.75 ± 20.13 | 220.85 ± 16.29 | 0.002 |
|  | LV EDV index (ml/m^2^) | 85.16 ± 9.70 | 106.06 ±7.82 | 0.002 |
|  | LV ESV (ml) | 71.69 ± 6.27 | 107.82 ± 14.79 | < 0.001 |
|  | LV ESV index (ml/m^2^) | 34.54 ± 3.02 | 51.78 ± 7.10 | < 0.001 |
|  | Stroke volume (ml) | 101.52 ± 13.19 | 108.55 ± 11.73 | 0.517 |
|  | LV EF (%) | 59.52 ± 2.21 | 49.75 ± 2.52 | < 0.001 |
|  | Cardiac output (L/min) | 7.70 ± 1.45 | 6.84 ± 0.94 | 0.621 |
| Female | LV mass (g) | 72.24 ± 13.94 | 88.88 ± 17.61 | 0.012^d^ |
|  | LV mass index (g/m^2^) | 38.97 ± 7.52 | 48.51 ± 9.61 | 0.008^d^ |
|  | LV EDV (ml) | 140.61 ± 18.04 | 183.50 ± 35.43 | < 0.001^d^ |
|  | LV EDV index (ml/m^2^) | 75.85 ± 9.73 | 100.16 ± 19.34 | 0.001^d^ |
|  | LV ESV (ml) | 53.00 ± 7.73 | 81.85 ± 10.27 | < 0.001 |
|  | LV ESV index (ml/m^2^) | 28.59 ± 4.17 | 44.67 ± 5.60 | < 0.001 |
|  | Stroke volume (ml) | 87.42 ± 11.46 | 91.74 ± 19.58 | 0.495^d^ |
|  | LV EF (%) | 62.23 ± 3.31 | 50.16 ± 6.91 | < 0.001^d^ |
|  | Cardiac output (L/min) | 5.66 ± 0.97 | 5.96 ± 1.89 | 0.615^d^ |

Values are presented as mean ± standard deviation or median ± median absolute deviation. n, number of patients; bpm, beats per minute; BMI, body mass index; BSA, body surface area; MR, magnetic resonance; LV, left ventricle; EDV, end diastolic volume; ESV, end systolic volume; EF, ejection fraction.

^a^P-Values by Mann-Whitney U test.

^b^Value is number of patients, with percentage in parentheses.

^c^P-Value by chi square test.

^d^P-Values by independent t-test.

**Table S2.** T1 coefficient of variance between observers in different left ventricular myocardial regions.

|  | |  | Patients with normal heart function | | | |  | Patients with abnormal heart function | | | |
| --- | --- | --- | --- | --- | --- | --- | --- | --- | --- | --- | --- |
|  | |  | CoV between two cardiac experts using | | CoV between two non-cardiac experts using | |  | CoV between two cardiac experts using | | CoV between two non-cardiac experts using | |
|  | | ns^a^ | Mean T1 | Median T1 | Mean T1 | Median T1 | ns^a^ | Mean T1 | Median T1 | Mean T1 | Median T1 |
| Global LV myocardium | | 416 | 5.73 | 4.95 | 4.90 | 4.44 | 496 | 4.49 | 3.65 | 4.21 | 3.71 |
| LAD | | 156 | 5.46 | 4.41 | 4.56 | 4.27 | 186 | 4.68 | 3.72 | 4.23 | 3.53 |
| RCA | | 130 | 5.41 | 4.76 | 3.04 | 2.78 | 155 | 4.08 | 3.27 | 3.33 | 2.91 |
| LCx | | 130 | 5.96 | 5.52 | 6.50 | 5.75 | 155 | 4.16 | 3.64 | 4.90 | 4.54 |
|  | |  |  |  |  |  |  |  |  |  |  |
| Basal | | 156 | 4.09 | 3.05 | 3.40 | 2.83 | 186 | 3.34 | 2.59 | 3.63 | 2.87 |
| 1 | Anterior | 26 | 5.10 | 2.90 | 3.14 | 2.48 | 31 | 4.71 | 3.61 | 5.67 | 3.80 |
| 2 | Anteroseptal | 26 | 2.42 | 2.68 | 2.42 | 1.67 | 31 | 2.18 | 1.69 | 2.57 | 2.03 |
| 3 | Inferoseptal | 26 | 3.04 | 2.64 | 2.09 | 2.41 | 31 | 2.00 | 1.14 | 2.43 | 2.47 |
| 4 | Inferior | 26 | 2.68 | 1.78 | 2.58 | 1.75 | 31 | 3.25 | 2.86 | 3.17 | 2.94 |
| 5 | Inferolateral | 26 | 1.63 | 1.25 | 3.31 | 2.34 | 31 | 2.34 | 1.83 | 3.41 | 2.53 |
| 6 | Anterolateral | 26 | 5.71 | 4.64 | 5.53 | 4.95 | 31 | 3.70 | 2.81 | 3.28 | 2.81 |
|  |  |  |  |  |  |  |  |  |  |  |  |
| Mid-ventricular | | 156 | 4.92 | 3.83 | 5.29 | 5.03 | 186 | 4.30 | 3.08 | 4.08 | 3.65 |
| 7 | Anterior | 26 | 4.95 | 4.14 | 6.42 | 6.82 | 31 | 6.19 | 4.64 | 4.87 | 4.59 |
| 8 | Anteroseptal | 26 | 3.76 | 2.37 | 3.97 | 3.53 | 31 | 2.53 | 1.61 | 4.05 | 3.31 |
| 9 | Inferoseptal | 26 | 1.64 | 1.38 | 1.52 | 1.15 | 31 | 2.11 | 1.31 | 2.19 | 1.42 |
| 10 | Inferior | 26 | 4.63 | 2.37 | 2.72 | 2.66 | 31 | 3.64 | 2.88 | 2.51 | 2.64 |
| 11 | Inferolateral | 26 | 3.07 | 2.66 | 6.05 | 5.75 | 31 | 2.94 | 2.35 | 3.90 | 3.05 |
| 12 | Anterolateral | 26 | 6.34 | 6.34 | 7.74 | 7.02 | 31 | 4.70 | 3.06 | 5.85 | 5.57 |
|  |  |  |  |  |  |  |  |  |  |  |  |
| Apical | | 104 | 8.21 | 7.76 | 5.78 | 5.20 | 124 | 6.04 | 5.40 | 5.12 | 4.78 |
| 13 | Anterior | 26 | 8.24 | 8.01 | 5.75 | 5.67 | 31 | 6.10 | 5.67 | 4.97 | 4.28 |
| 14 | Septal | 26 | 2.69 | 2.20 | 3.27 | 2.47 | 31 | 3.26 | 2.61 | 2.01 | 2.27 |
| 15 | Inferior | 26 | 9.57 | 9.07 | 4.10 | 4.05 | 31 | 6.77 | 5.57 | 5.26 | 4.32 |
| 16 | Lateral | 26 | 8.93 | 8.67 | 8.47 | 7.40 | 31 | 5.64 | 6.05 | 6.87 | 6.95 |

Data are in percentage. AHA, American Heart Association; ns, number of segments; CoV, coefficient of variance.

^a^ The number of segments reflects six combinations of segment comparisons between two observers.

**Table S3.** Shapiro-Wilk normality testing of native T1 segments in different left ventricular myocardial regions using mean quantification of pixel-wise values.

|  |  |  | Patients with normal heart function |  | Patients with abnormal heart function |
| --- | --- | --- | --- | --- | --- |
|  |  | ns^a^ | P-Value^b^ of native T1 | ns^a^ | P-Value^b^ of native T1 |
| Global LV myocardium | | 2496 | <0.001 | 2976 | <0.001 |
| LAD | | 936 | <0.001 | 1116 | <0.001 |
| RCA | | 780 | <0.001 | 930 | <0.001 |
| LCx | | 780 | <0.001 | 930 | <0.001 |
|  | |  |  |  |  |
| Basal | | 936 | <0.001 | 1116 | <0.001 |
| 1 | Anterior | 156 | >0.05 | 186 | <0.001 |
| 2 | Anteroseptal | 156 | <0.001 | 186 | <0.001 |
| 3 | Inferoseptal | 156 | <0.001 | 186 | <0.001 |
| 4 | Inferior | 156 | <0.01 | 186 | <0.001 |
| 5 | Inferolateral | 156 | <0.05 | 186 | <0.001 |
| 6 | Anterolateral | 156 | <0.001 | 186 | <0.001 |
|  |  |  |  |  |  |
| Mid-ventricular | | 936 | <0.001 | 1116 | <0.001 |
| 7 | Anterior | 156 | >0.05 | 186 | <0.001 |
| 8 | Anteroseptal | 156 | <0.01 | 186 | <0.001 |
| 9 | Inferoseptal | 156 | <0.001 | 186 | <0.001 |
| 10 | Inferior | 156 | <0.001 | 186 | <0.001 |
| 11 | Inferolateral | 156 | <0.01 | 186 | <0.001 |
| 12 | Anterolateral | 156 | <0.001 | 186 | <0.001 |
|  |  |  |  |  |  |
| Apical | | 624 | <0.001 | 744 | <0.001 |
| 13 | Anterior | 156 | <0.001 | 186 | <0.001 |
| 14 | Septal | 156 | <0.01 | 186 | <0.001 |
| 15 | Inferior | 156 | <0.001 | 186 | 0.056 |
| 16 | Lateral | 156 | <0.001 | 186 | <0.001 |

^a^ The number of segments reflects six combinations of segment comparisons between four observers.

^b^ P-Value of <0.05 is considered as statistically significantly different from normal distribution.

**Table S4.** Shapiro-Wilk normality testing of native T1 segments in different left ventricular myocardial regions with using median quantification of pixel-wise values.

|  |  |  | Patients with normal heart function |  | Patients with abnormal heart function |
| --- | --- | --- | --- | --- | --- |
|  |  | ns^a^ | P-Value^b^ of native T1 | ns^a^ | P-Value^b^ of native T1 |
| Global LV myocardium | | 2496 | <0.001 | 2976 | <0.001 |
| LAD | | 936 | <0.001 | 1116 | <0.001 |
| RCA | | 780 | <0.001 | 930 | <0.001 |
| LCx | | 780 | <0.001 | 930 | <0.001 |
|  | |  |  |  |  |
| Basal | | 936 | <0.001 | 1116 | <0.001 |
| 1 | Anterior | 156 | <0.01 | 186 | <0.001 |
| 2 | Anteroseptal | 156 | <0.001 | 186 | <0.001 |
| 3 | Inferoseptal | 156 | <0.001 | 186 | <0.001 |
| 4 | Inferior | 156 | <0.001 | 186 | <0.001 |
| 5 | Inferolateral | 156 | <0.01 | 186 | <0.001 |
| 6 | Anterolateral | 156 | <0.001 | 186 | <0.001 |
|  |  |  |  |  |  |
| Mid-ventricular | | 936 | <0.001 | 1116 | <0.001 |
| 7 | Anterior | 156 | 0.01 | 186 | <0.001 |
| 8 | Anteroseptal | 156 | >0.05 | 186 | <0.001 |
| 9 | Inferoseptal | 156 | <0.001 | 186 | <0.001 |
| 10 | Inferior | 156 | <0.001 | 186 | <0.001 |
| 11 | Inferolateral | 156 | <0.01 | 186 | <0.001 |
| 12 | Anterolateral | 156 | <0.001 | 186 | <0.001 |
|  |  |  |  |  |  |
| Apical | | 624 | <0.001 | 744 | <0.001 |
| 13 | Anterior | 156 | <0.001 | 186 | <0.001 |
| 14 | Septal | 156 | 0.01 | 186 | <0.001 |
| 15 | Inferior | 156 | <0.001 | 186 | <0.001 |
| 16 | Lateral | 156 | <0.001 | 186 | <0.001 |

^a^ The number of segments reflects six combinations of segment comparisons between four observers.

^b^ P-Value of <0.05 is considered as statistically significantly different from normal distribution.

**Table S5.** Native T1 segments on different left ventricular myocardial regions of all patients using mean quantification of pixel-wise values.

|  | |  | T1 value of all patients evaluated by QMASS | T1 value of all patients evaluated by MATLAB |  |
| --- | --- | --- | --- | --- | --- |
|  | | n | Mean ± SD (msec) | Mean ± SD (msec) | P-Value ^a^ |
| Basal | |  |  |  |  |
| 1 | Anterior | 57 | 1017.00 ± 37.45^b^ | 980.35 ± 36.49^b^ | 0.001^c^ |
| 2 | Anteroseptal | 57 | 1028.33 ± 58.81 | 990.69 ± 55.12 | 0.001 |
| 3 | Inferoseptal | 57 | 1027.57 ± 57.91 | 989.47 ± 54.29 | <0.001 |
| 4 | Inferior | 57 | 1032.91 ± 58.07 | 990.50 ± 55.27 | <0.001 |
| 5 | Inferolateral | 57 | 1006.09 ± 58.41 | 966.21 ± 53.68 | <0.001 |
| 6 | Anterolateral | 57 | 999.04 ± 52.72 | 962.32 ± 49.58 | <0.001 |
| Mid-ventricular | |  |  |  |  |
| 7 | Anterior | 57 | 1002.31 ± 41.29^b^ | 965.37 ± 40.59^b^ | <0.01^c^ |
| 8 | Anteroseptal | 57 | 1004.93 ± 41.73^b^ | 969.38 ± 40.12^b^ | 0.001^c^ |
| 9 | Inferoseptal | 57 | 1019.10 ± 57.72 | 982.28 ± 54.35 | 0.001 |
| 10 | Inferior | 57 | 1023.45 ± 40.00^b^ | 987.55 ± 36.29^b^ | 0.001^c^ |
| 11 | Inferolateral | 57 | 997.31 ± 57.63 | 963.86 ± 56.02 | <0.01 |
| 12 | Anterolateral | 57 | 987.60 ± 66.65 | 952.63 ± 62.74 | <0.01 |
| Apical | |  |  |  |  |
| 13 | Anterior | 57 | 990.68 ± 87.32 | 954.51 ± 83.38 | <0.05 |
| 14 | Septal | 57 | 1018.12 ± 75.02 | 980.62 ± 70.06 | <0.01 |
| 15 | Inferior | 57 | 969.88 ± 111.76 | 935.01 ± 100.70 | >0.05 |
| 16 | Lateral | 57 | 981.72 ± 103.28 | 945.80 ± 98.62 | >0.05 |

SD, standard deviation; n, number of patients.

^a^ P-Values of T1 comparison evaluated by QMASS and MATLAB groups were made using independent sample t-test.

^b^ Values are presented as median ± median absolute deviation.

^c^ P-Values of T1 comparison evaluated by QMASS and MATLAB groups were made using independent Mann-Whitney U test.
